# Pyroptosis Plays a Key Role Through Macrophages in Primary Biliary Cholangitis of Mice

**DOI:** 10.1155/bmri/5563223

**Published:** 2026-02-26

**Authors:** Lin-Xiang Huang, Zi-Long Wang, Xiao-Xiao Wang, Zi-xuan Qiu, Jia-rui Zheng, Rui Jin, Bo Feng

**Affiliations:** ^1^ Peking University Hepatology Institute, Infectious Disease and Hepatology Center of Peking University People′s Hospital, Beijing Key Laboratory of Hepatitis C and Immunotherapy for Liver Diseases, Beijing International Cooperation Base for Science and Technology on NAFLD Diagnosis, Peking University People′s Hospital, Beijing, China, pku.edu.cn

**Keywords:** autoimmune liver diseases, caspase-1, cholestatic diseases, gasdermin D, macrophages, NLRP3, primary biliary cholangitis, pyroptosis

## Abstract

**Background and Aims:**

Primary biliary cholangitis (PBC) is an autoimmune intrahepatic cholestatic disease with both environmental and genetic participation. In this study, we aim to investigate the involvement of pyroptosis in PBC mice based on prior bioinformatic analysis of PBC patients and explore the immune cell populations potentially involved.

**Methods:**

KEGG pathway enrichment analysis was performed using the GSE119600 dataset from the Gene Expression Omnibus (GEO) database, which includes whole‐blood samples from PBC patients (*n* = 90) and non–liver disease CTRs (*n* = 47). The bioinformatic analysis was conducted prior to and in guidance of the subsequent animal experiments. A total of 20 female C57BL/6 mice aged 4–6 months were randomly divided into the PBC group and the control (CTR) group. The PBC model was induced by two doses of 2‐nonynoic acid (2OA‐BSA) and polyinosinic–polycytidylic acid (poly I: C) for a total of 12 weeks. The pyroptosis pathway was examined by quantitative real‐time PCR (qRT‐PCR), Western blotting, and immunohistochemistry. Immunofluorescent (IF) staining and flow cytometry were performed on liver samples from PBC mice to assess the pyroptosis pathway and distinct immune cell populations.

**Results:**

Toll‐like receptor and NOD‐like receptor signaling pathways were identified in blood samples of PBC patients in KEGG pathway enrichment analysis. In the PBC mouse model, the pyroptosis pathway was found to be upregulated by qRT‐PCR (*p* < 0.05) and Western blotting (0.7786 ± 0.1371, *p* < 0.001). IHC staining revealed increased GSDMD and Casp1 expression in PBC mice, and macrophages were identified as the main cell type expressing gasdermin D (GSDMD) by IF staining. Flow cytometry showed a decrease in the percentage of M2 macrophages (6.10 ± 2.12 vs. 3.24 ± 0.93, *p* < 0.05).

**Conclusions:**

Pyroptosis plays a key role in PBC patients and 2OA‐BSA induced PBC mice, with macrophages possibly serving as important executors. Inhibition of the pyroptosis pathway might be a potential target for the future treatment of PBC.

## 1. Introduction

Primary biliary cholangitis (PBC) is a progressive, nonsuppurative, destructive intrahepatic cholestatic disease, which is considered to be an immunological disorder with both environmental and genetic involvement [1]. Ursodeoxycholic acid (UDCA), a hydrophilic bile acid, is currently the first‐line treatment for PBC by regulating the metabolism of bile acids [2]. However, around 40% of patients with PBC have a poor response to UDCA, causing early hepatic decompensation, cirrhosis, and finally requiring liver transplantation [3], leading to a poor prognosis and decreased long‐term survival rate [4, 5].

Bile acids could activate the formation of reactive oxygen species (ROS) and play a key role in the pathogenesis of PBC [6]. In these patients, the ratio of conjugated/unconjugated bile acids increases, which has a toxic effect on hepatic and biliary cells [7]. It is reported that bile acids could affect the activation of the NLRP3 inflammasome, which belongs to the family of nucleotide‐binding and oligomerization domain‐like receptors (NOD‐like receptors, NLRs) [8]. NLRP3 is an important molecule in the process of pyroptosis—a newly discovered cell death pathway characterized by gasdermin‐mediated programmed necrosis [9]. Pyroptosis has been shown to be involved in many diseases including immunity, cancer, and beyond [10], which is usually triggered by pathogen attack or tissue injury and serves to eliminate pathogens via cell lysis, which can subsequently trigger local or systemic inflammation [11]. This process is generally divided into the caspase‐1 (Casp1)‐dependent canonical pathway and the Casp11/4/5‐dependent noncanonical pathway, with gasdermin D (GSDMD) being the terminal executor [12, 13], which releases gasdermin‐N domain and perforates the plasma membrane. Notably, the toll‐like receptors (TLRs) pathway is involved in the classical pathway [14].

It is well‐studied that pyroptosis participates in the pathogenesis of many liver diseases, including steatohepatitis, alcoholic liver disease, viral hepatitis, and so on. [14]. In steatohepatitis, GSDMD has been proven to be an important executor in its pathogenesis by controlling cytokine secretion, NF‐ĸB pathway activation, and lipogenesis [15]. In a previous study, pyroptotic molecules are reported to be highly expressed in cholestatic liver diseases in human and mouse models, and statistical analysis revealed that NLRP3, which activates Casp1 by cleaving pro‐Casp1 protein, is significantly increased in the liver tissue of PBC patients [16], although the specific mechanisms and relative animal studies remain unclear. Previous studies have reported conflicting results about the effects of bile acids on the activation of NLRP3 inflammasome in PBC [17, 18], therefore, whether cholestasis would promote or inhibit the process of pyroptosis requires further investigation. In this study, we aim to investigate the involvement of pyroptosis in PBC mice based on prior bioinformatic analysis of PBC patients and explore the immunological cell components possibly involved.

## 2. Materials and Methods

### 2.1. Data Sources and Differentially Expressed Gene (DEG) Analysis

The RNA‐sequencing data from the GSE119600 dataset were obtained from the Gene Expression Omnibus (GEO) database (https://www.ncbi.nlm.nih.gov/geo/). We performed pathway enrichment analysis (Kyoto Encyclopedia of Genes and Genomes, KEGG) on the GSE119600 dataset using the “ClusterProfiler” R package, with a significance threshold set at a *p* value of less than 0.05.

### 2.2. Mouse Model

Four‐ to six‐month‐old female C57BL/6 mice were purchased from Beijing Weitong Lihua Experimental Animal Technology (Beijing, China) and kept in a specific pathogen‐free (SPF) facility at Peking University People′s Hospital, maintained at 25°C–30°C with a 12‐hour light/dark cycle. All animal experiments were approved by the Ethics Committee of Peking University People′s Hospital.

The mice were assigned to two groups: the PBC group and the control (CTR) group. PBC was induced in the mice by administering two injections of 2‐nonynoic acid (2OA‐BSA) (100 *μ*l/mouse) at 2‐week intervals. The first injection was prepared by mixing 1 mg of 2OA‐BSA with 500 *μ*l PBS, emulsified with 500‐*μ*l complete Freund′s adjuvant (CFA), and the second injection was prepared using the same dose but with incomplete Freund′s adjuvant (IFA). Polyinosinic‐polycytidylic acid (poly I: C) was administered twice a week. The CTR received PBS (100 *μ*l/mouse) at the same frequency as the PBC group. Mice were sacrificed after 12 weeks, with euthanasia performed by cervical dislocation. Blood and liver samples were collected (Figure S1).

### 2.3. Immunological Examination

The levels of anti‐PDC‐E2 antibody were quantified using enzyme‐linked immunosorbent assay (ELISA) kits (MEIMIAN #46349 M1). The diluted antibodies and sera were incubated for 1.5 h and then mixed with horseradish peroxidase (HRP)‐conjugated streptavidin at room temperature. After incubation, substrate solution was added to each well, and absorbance was measured at 450 nm using a spectrophotometer.

### 2.4. Quantitative Real‐Time PCR (qRT‐PCR)

Total RNA was extracted from fresh liver tissues using TRIzol reagent according to the manufacturer′s instructions and treated with DNase (QIAGEN) to remove genomic DNA contamination. Due to limited tissue availability after sample processing, qRT‐PCR was performed on liver samples from individual mice (CTR, *n* = 5; PBC, *n* = 6). RNA concentration was assessed using the NanoDrop One/OneC Microvolume UV‐Vis Spectrophotometer (Thermo Fisher Scientific). cDNA synthesis was performed using the SuperScript First‐Strand Synthesis System (Bio‐Rad). Transcript relative gene expression levels were measured by qRT‐PCR using SYBR Green (Bio‐Rad) on the CFX Real‐Time PCR Detection System (Bio‐Rad). Gene expression levels were normalized to GAPDH as an internal reference. The sequences of the primer pairs are listed in Table 1.

**Table 1:** Sequences of primer pairs in qRT‐PCR of mice.

Gene	Forward primer (5 ^′^–3 ^′^)	Reverse primer (5 ^′^–3 ^′^)
NLRP3	TCACAACTCGCCCAAGGAGGAA	AAGAGACCACGGCAGAAGCTAG
Caspase‐1	AAGAAACGCCATGGCTGACAA	TCACATAGGTCCCGTGCCTT
GSDMD	GGTGCTTGACTCTGGAGAACTG	GCTGCTTTGACAGCACCGTTGT

### 2.5. Western Blotting

Liver tissues were lysed in RIPA buffer (R&D) supplemented with a protease inhibitor (Thermo Fisher Scientific) and a phosphatase inhibitor cocktail (Thermo Fisher Scientific). Analyses were conducted on samples from 10 mice (*n* = 5 per group) due to limited tissue availability after sample processing. Protein concentration was measured using the BCA Protein Assay Kit (Pierce), and equal amounts of protein were separated by SDS‐PAGE and transferred onto nitrocellulose membranes. The membranes were blocked with 5% nonfat milk at room temperature for 1 h and then incubated overnight with primary antibodies: anti‐mouse GAPDH (Abcam #ab181602) and anti‐mouse GSDMD (Abcam #ab219800). After washing, membranes were incubated with the corresponding HRP‐conjugated secondary antibodies. Protein bands were visualized using enhanced chemiluminescence (ECL) and imaged with the ChemiDocTouch Imaging System (Bio‐Rad). Due to the large number of mouse samples, proteins were separated on two polyacrylamide gels run simultaneously under identical electrophoresis and transfer conditions. The same antibody solutions, exposure times, and imaging parameters were used for both blots to ensure comparability.

### 2.6. Hematoxylin and Eosin (H&E) Staining and Immunohistochemistry (IHC) Staining

H&E staining was performed on paraffin‐embedded liver sections, followed by dewaxing, rehydration, and staining. Images were captured using an inverted microscope. Histological staging was classified into four categories and evaluated semiquantitatively according to the study reported by Tsuneyama et al. [19]. For IHC staining, liver sections were incubated overnight at 4°C with primary antibodies against GSDMD (Abcam #ab219800), followed by incubation with secondary antibodies (ZSGB‐BIO, China) for 30 min at 37°C.

### 2.7. Immunofluorescent (IF) Staining

Liver tissue sections were blocked with 5% skim milk for 1 h at 37°C, followed by incubation with primary antibodies (CK19, CD4, and GSDMD) and nuclear counterstaining with DAPI at 4°C overnight. Sections were washed with TBS‐0.3% Tween‐20 (TBST) and incubated with secondary antibodies at room temperature for 2 h. Antibodies used in IF staining are listed in Table S1.

### 2.8. Flow Cytometry

Flow cytometry analysis of macrophages was performed on mononuclear cells (MNCs) isolated from liver samples of individual mice (CTR, *n* = 10; PBC, *n* = 10). Single‐cell suspensions were prepared after liver tissue was harvested and purified by Percoll density gradient centrifugation. Cell viability was assessed by excluding dead cells using forward and side scatter gating, as it was assessed using a viability dye, confirming that freshly isolated cells consistently exhibited high viability (> 90%). Approximately 1 × 10^6^ cells were stained with fluorochrome‐conjugated antibodies against CD45, CD11b, F4/80, Ly6G, CD11c, and CD206 in the dark. Gating thresholds for CD11c and CD206 were set using negative and single‐stained CTRs. M1 macrophages were defined as F4/80 + CD11b + CD11c+, whereas M2 macrophages were identified as F4/80 + CD11b + CD206+ [20, 21]. The antibodies used in flow cytometry are listed in Table S2.

### 2.9. Statistical Analysis

All data were analyzed using independent‐samples *t*‐tests in IBM SPSS Statistics 27.0 and presented as mean ± standard deviation (SD). A *p* value < 0.05 was considered statistically significant.

## 3. Results

### 3.1. TLR and NOD‐Like Receptor Signaling Pathway Were Identified in Blood Samples of PBC Patients

**Figure 1:**
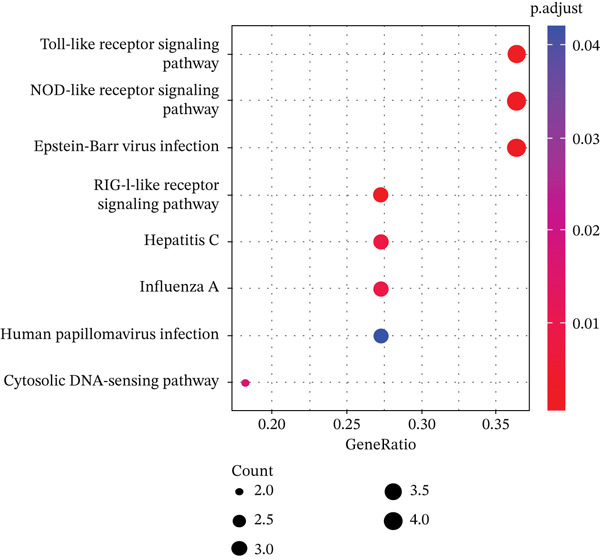
KEGG pathway enrichment analysis.

KEGG pathway enrichment analysis (Figure 1) was performed on GSE119600 dataset, including whole blood samples from PBC patients (*n* = 90) and non–liver disease CTRs (*n* = 47). Toll‐like receptor and NOD‐like receptor signaling pathways were identified as among the most significant, which are key components involved in the canonical pyroptosis pathway as we described above.

### 3.2. Immunological and Histological Examination Proves the Success of PBC Mouse Modeling

During modeling, there were no significant differences in the general condition of mice, including food and water intake, body weight (Figure 2a), activity, and coat color. No signs of jaundice were observed in the PBC mouse model. After injection of 2OA‐BSA and poly I: C, serum anti‐PDC‐E2 antibodies were detected in the PBC mice (Figure 2b), and the validity of the assay was confirmed by the inclusion of standard positive and negative CTRs. Histological examination (Figure 2c) further revealed marked inflammatory infiltration around the portal tracts, accompanied by bile duct destruction and ductular proliferation in the PBC mouse model (1.03 ± 0.17, *p* < 0.0001).

Figure 2The general performance of the PBC mouse model. (a) The changes in body weight of mice at 4‐12 weeks after modeling in CTR and PBC mice, (b) detection and quantification of anti‐PDC‐E2 antibody in sera of CTR and PBC mice by using ELISA, and (c) H&E pathological observation of the liver after modeling.  ^∗^ *p* < 0.05,  ^∗∗^ *p* < 0.01,  ^∗∗∗^ *p* < 0.001,  ^∗∗∗∗^ *p* < 0.0001.

**Figure:**
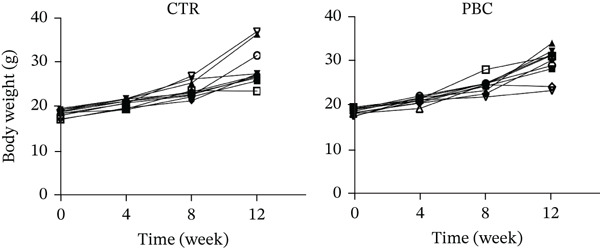
(a)

**Figure:**
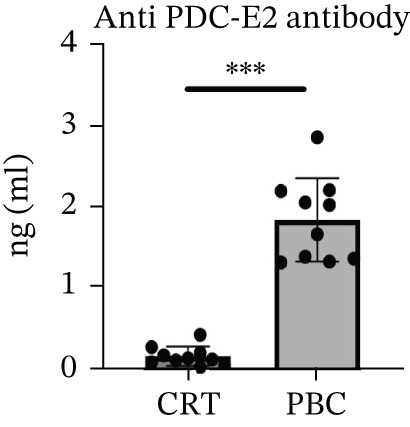
(b)

**Figure:**
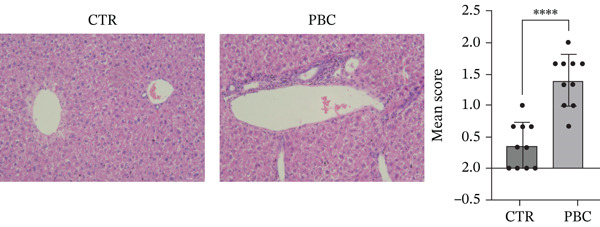
(c)

### 3.3. GSDMD is Upregulated in Liver Tissues of 2OA‐Induced PBC Mice

To verify the expression levels of GSDMD in the 2OA‐induced PBC mice, we evaluated the mRNA and protein levels of GSDMD from liver tissues. Both the mRNA and protein (0.7786 ± 0.1371, *p* < 0.001) levels of GSDMD from PBC mice significantly increased compared with that of normal CTRs, determined by qRT‐PCR (Figure 3a) and Western blotting (Figure 3b), respectively. Western blots were performed on two separate gels due to the large number of mouse samples. All samples were processed under identical experimental conditions. The blots shown were aligned for presentation. Full‐length gels of Western blotting are presented in Figure S2. Additionally, qRT‐PCR analysis revealed that the expression levels of all key elements of the classic pyroptosis pathway (i.e., NLRP3‐Casp1‐GSDMD) were significantly higher than CTR (*p* < 0.05). IHC staining also revealed increased GSDMD (Figure 3c) expression in PBC mice, especially around the portal area, suggesting that pyroptosis is involved in the pathogenesis of 2OA‐induced PBC mice.

Figure 3GSDMD is upregulated in liver tissues of 2OA‐induced PBC mouse model. (a) Hepatic mRNA levels of NLRP3‐Caspase‐1‐GSDMD pathway and (b) hepatic protein expression of GSDMD. The PBC group was cropped from different gels due to length limitation, indicated with white space. (c) Representative immunohistochemistry stained sections of GSDMD and Caspase‐1 in control and PBC mouse model.  ^∗^ *p* < 0.05,  ^∗∗^ *p* < 0.01,  ^∗∗∗^ *p* < 0.001.

**Figure:**
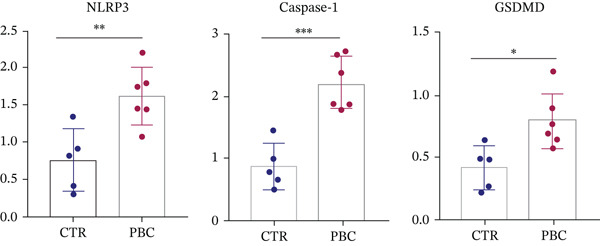
(a)

**Figure:**
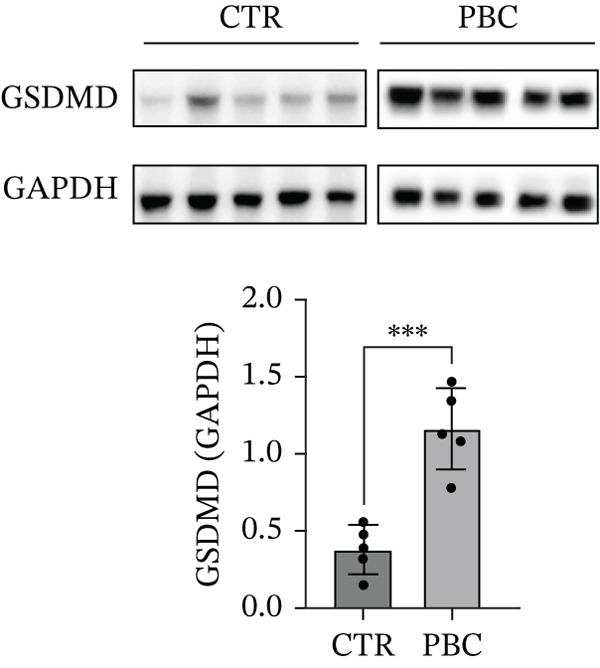
(b)

**Figure:**
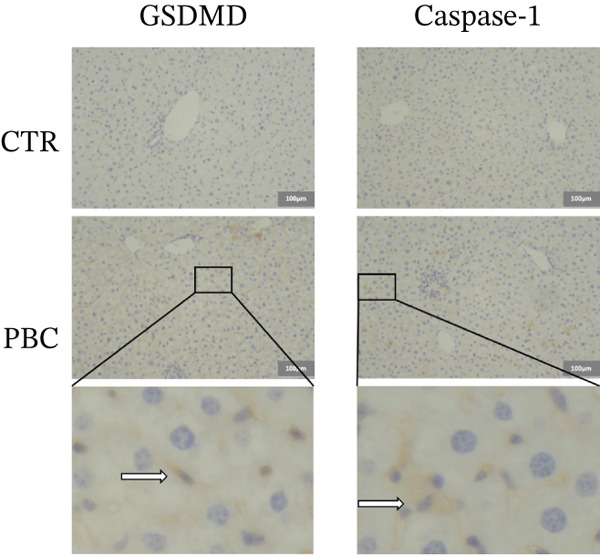
(c)

### 3.4. Changes of M1/M2 Macrophage Ratio in Liver Samples of PBC Mice

To investigate the role of macrophages in PBC pathogenesis, we performed flow cytometry on PBC mouse model and CTR group. No significant differences were observed between the two groups in the percentage of M1 macrophages (0.83 ± 0.46 vs. 0.81 ± 0.42, *p* > 0.05) and M1/M2 macrophage ratio (0.17 ± 0.13 vs. 0.30 ± 0.24, *p* > 0.05), whereas compared with CTR, PBC mice had a significantly lower level of the percentage of M2 macrophages (6.10 ± 2.12 vs. 3.24 ± 0.93, *p* < 0.05) in the liver (Figure 4).

Figure 4The M1/M2 macrophage ratio in liver samples of PBC mice. (a) Gating strategy of flow cytometry in control group, (b) gating strategy of flow cytometry in PBC mouse model group, and (c) the differences in the percentage of M1 and M2 macrophages and M1/M2 macrophage ratio.  ^∗^ *p* < 0.05,  ^∗∗^ *p* < 0.01.

**Figure:**
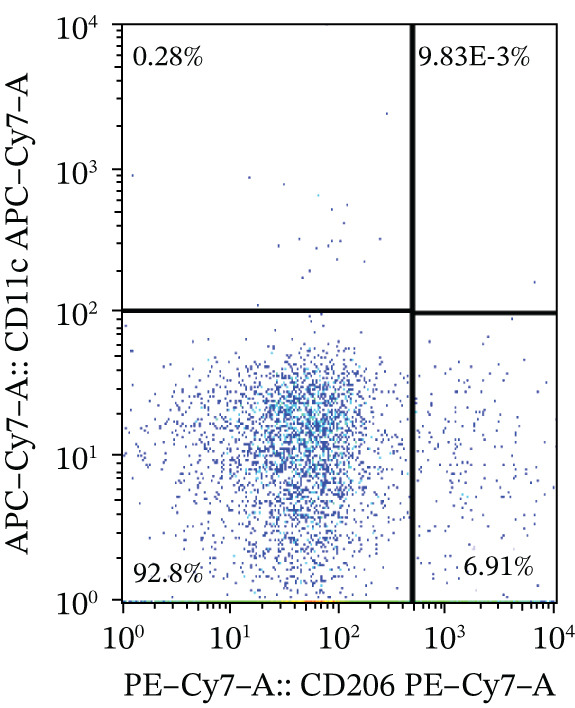
(a)

**Figure:**
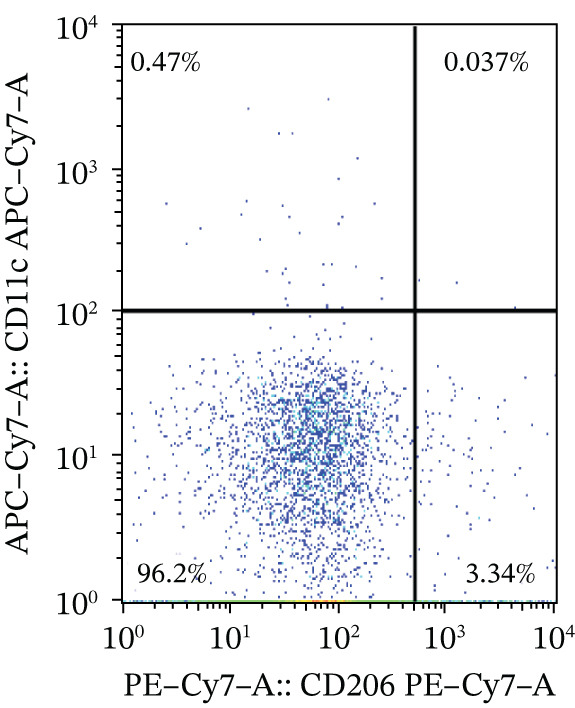
(b)

**Figure:**
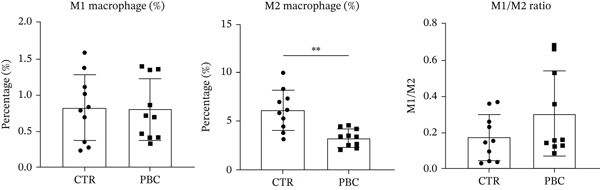
(c)

### 3.5. Pyroptosis Mainly Happens in Macrophages in the Liver of PBC Mice

To identify the main cell type expressing GSDMD during the pathogenesis of PBC, we examined the expression of DAPI, CK19, CD4, F4/80, and GSDMD in the liver tissue specimens. Multiplex immunofluorescence (mIF) revealed higher GSDMD expression in the liver tissues of PBC mice compared with CTR. Notably, GSDMD was predominantly colocalized with F4/80+ macrophages in PBC mice, whereas little colocalization was observed with DAPI+ nuclei, CK19+ cholangiocytes, or CD4+ T lymphocytes (Figure 5).

Figure 5(a, b) Multiplex immunofluorescence analysis of the expression levels of DAPI, CK19, CD4, F4/80, and GSDMD in PBC and CTR group.

**Figure:**
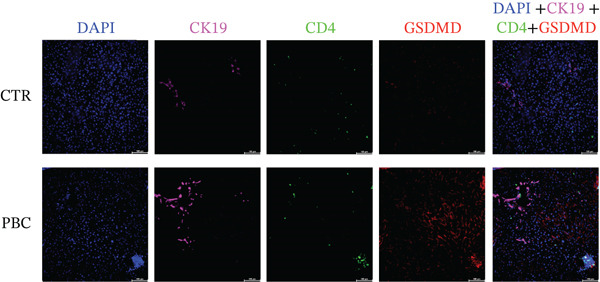
(a)

**Figure:**
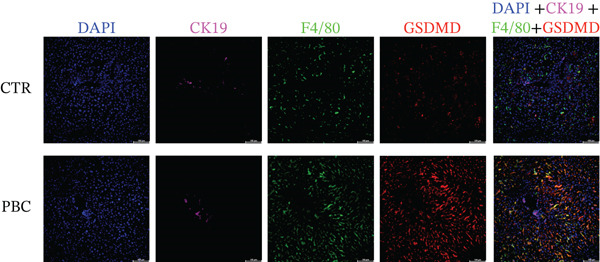
(b)

## 4. Discussion

In this study, bioinformatic analysis of data from the GEO database suggested that the pyroptosis pathway was possibly involved in the pathogenesis of PBC patients. To further explore this finding, a 2OA‐BSA–induced PBC mouse model was introduced. The results showed that PBC mice had a significantly higher expression of canonical pyroptosis pathway than CTR, with macrophages identified as the main cell type involved. Additionally, the M1/M2 macrophage ratio increased in PBC mice after modeling, providing new insights into the pathogenesis of PBC and potential therapeutic strategies.

NF‐*κ*B is a transcription factor involved in the regulation of cell signaling and inflammatory responses. It also plays a crucial role in promoting the classical activation of macrophages [22, 23]. Recent studies have shown that NF‐*κ*B is a main executor in the canonical pathway of pyroptosis [14], which provides a possibility for macrophages to participate in the bioprocess of pyroptosis.

In the KEGG pathway enrichment analysis performed on whole blood samples of PBC patients, we found that TLR and NLR signaling pathways were enriched as the top 2 pathways. In a study of ozone exposure causing lung injury in rats, researchers demonstrated that endogenous DAMPs can be released after lung injury, which interact with TLR2 and TLR4 to activate the downstream NF‐*κ*B signaling pathway and further activate the NLRP3 inflammasome complex to regulate the ozone‐induced pyroptosis [24]. Related studies found that PBC induced the release of inflammatory factors by activating the TLR4/MyD88/NF‐*κ*B signaling pathway, which results in the liver damage in PBC mice [25, 26]. These results suggest that pyroptosis could be an important process in the pathogenesis of PBC, with macrophages being the possible effector cells.

In our study, we proved that all key elements of the classic pyroptosis pathway NLRP3‐Casp1‐GSDMD significantly increased in mRNA in the PBC mouse model, consistent with previous observations in cholestatic patients [16], providing direct evidence that inflammasome‐mediated pyroptosis is activated during PBC‐related cholestatic liver injury. These findings highlight the pivotal role of the NLRP3‐Casp1‐GSDMD axis in disease progression. Interestingly, previous studies have reported conflicting effects of bile acids on NLRP3 activation. Some demonstrated that bile acids, particularly lithocholic acid (LCA), inhibit inflammasome activation via TGR5‐cAMP‐PKA signaling, promoting NLRP3 phosphorylation and ubiquitination [17]. In contrast, other bile acids such as chenodeoxycholic acid (CDCA) and deoxycholic acid (DCA) can activate both Signal 1 and 2 of the NLRP3 inflammasome in inflammatory macrophages, often through calcium influx, especially under sepsis or high‐inflammatory conditions [18]. These discrepancies likely reflect differences in cell types, bile acid species and concentrations, and pathophysiological context, suggesting that bile acids exert highly context‐dependent regulation of NLRP3 inflammasome activation.

Additionally, in this study, we found that the expression of GSDMD colocalizes with F4/80+ macrophages in the PBC mouse model. It is reported that M1 macrophages have the pro‐inflammatory, pathogen‐killing property, whereas M2 macrophages have the ability to promote cell proliferation and tissue repair [27]. In vitro, M1 macrophages are induced by infectious microorganism‐related molecules and/or inflammation‐related cytokines (TNF‐*α* or IFN‐*γ*) [28]. These cells produce toxic effector molecules (ROS and NO) and inflammatory cytokines (IL‐1*β*, TNF, and IL‐6), participate in polarized Th1 responses and help combat intracellular parasites and tumors. Conversely, macrophages polarize into M2 macrophages upon stimulation with IL‐4 or IL‐13, executing anti‐inflammatory and prohealing functions [29]. In our experiment, the IF showed colocalization of GSDMD with F4/80+ macrophages in the liver tissues of PBC mice, and flow cytometry revealed a significantly lower proportion of M2 macrophages in PBC mice. A tendency of increase in the M1/M2 macrophage ratio was observed in the PBC group. However, no significant result was obtained.

Our study for the first time proposed the involvement of pyroptosis in the pathogenesis of PBC and highlighted the potential role of macrophages in this disease. Nevertheless, there are several limitations. Firstly, due to the low incidence of PBC and liver biopsy not being routinely performed for its diagnosis, we can only perform the series of experiments on the 2OA‐BSA–induced mouse model, which limits the strength of validation. Human PBC liver validation is intended to be explored in future work, which would provide more direct and convincing evidence of the translational relevance of our findings. Secondly, our mouse model can only partially mimic the pathogenesis of PBC. A better PBC mouse model still needs to be discovered. Finally, due to limited liver tissue and time/resources, functional validation experiments such as LDH release assays and GSDMD knockdown could not be performed in this study. For future studies, we plan to carry out these validations in vitro and in GSDMD^−/−^ knockout mice to further confirm the functional role of macrophage pyroptosis. Investigating macrophage biological behavior and the underlying gene‐level mechanisms in PBC will be the focus of our next studies.

## 5. Conclusions

In this study, we demonstrated that pyroptosis was involved in the liver tissue of 2OA‐BSA induced PBC mice, with macrophages possibly serving as an important executor. Inhibition of the pyroptosis pathway might be a potential therapeutic target for PBC.

## Author Contributions

All authors contributed to the study conception and design. Material preparation, data collection and analysis were performed by Zi‐xuan Qiu, Jia‐rui Zheng, and Rui Jin. Animal experiments were performed by Xiao‐Xiao Wang, Zi‐Long Wang, and Lin‐Xiang Huang. The first draft of the manuscript was written by Lin‐Xiang Huang, Zi‐Long Wang, Bo Feng, and all authors commented on previous versions of the manuscript. Lin‐Xiang Huang and Zi‐Long Wang contribute equally to this article.

## Funding

No funding was received for this manuscript.

## Disclosure

All authors read and approved the final manuscript.

## Ethics Statement

This study was approved by the Ethics Committee of Peking University People′s Hospital (2020PHE081). The study followed ARRIVE guidelines (http://www.nc3rs.org.uk/arrive-guidelines). All institutional and national guidelines for the care and use of laboratory animals were followed.

## Consent

The authors have nothing to report.

## Conflicts of Interest

The authors declare no conflicts of interest.

## Supporting information


**Supporting Information** Additional supporting information can be found online in the Supporting Information section. **Figure S1:** The establishment of PBC mouse model and study design. **Figure S2:** Full‐length gels of Western blotting. **Table S1:** Antibodies used in immunofluorescent staining. **Table S2:** Antibodies used in flow cytometric analysis.

## Data Availability

The datasets used and/or analyzed during the current study are available from the corresponding author on reasonable request.
