# Supplementary material for: Pyroptosis Plays a Key Role Through Macrophages in Primary Biliary Cholangitis of Mice
Source: Biomed Res Int. 2026 Feb 26;2026:5563223. doi: 10.1155/bmri/5563223 (PMC12942079; doi:10.1155/bmri/5563223)
Supplement: Supplementary file 1 — Supporting Information Additional supporting information can be found online in the Supporting Information section. Figure S1: The establishment of PBC mouse model and study design. Figure S2: Full‐length gels of Western blotting. Table S1: Antibodies used in immunofluorescent staining. Table S2: Antibodies used in flow cytometric analysis. [file BMRI-2026-5563223-s001.docx]

Supplementary Material

This file includes:

Fig.S1. The establishment of PBC mouse model and study design.

Fig.S2. Full-length gels of western blotting.

Table.S1 Antibodies Used in Immunofluorescent staining.

Table.S2 Antibodies Used in Flow Cytometric Analysis.

Fig.S1. The establishment of PBC mouse model and study design.


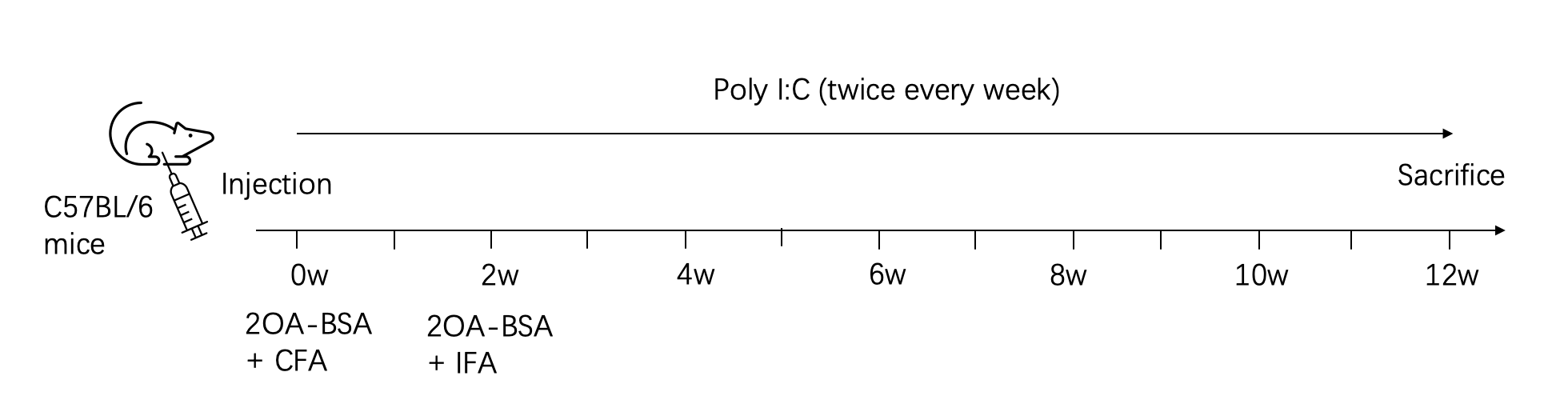


Fig.S2. Full-length gels of western blotting in Fig.3B


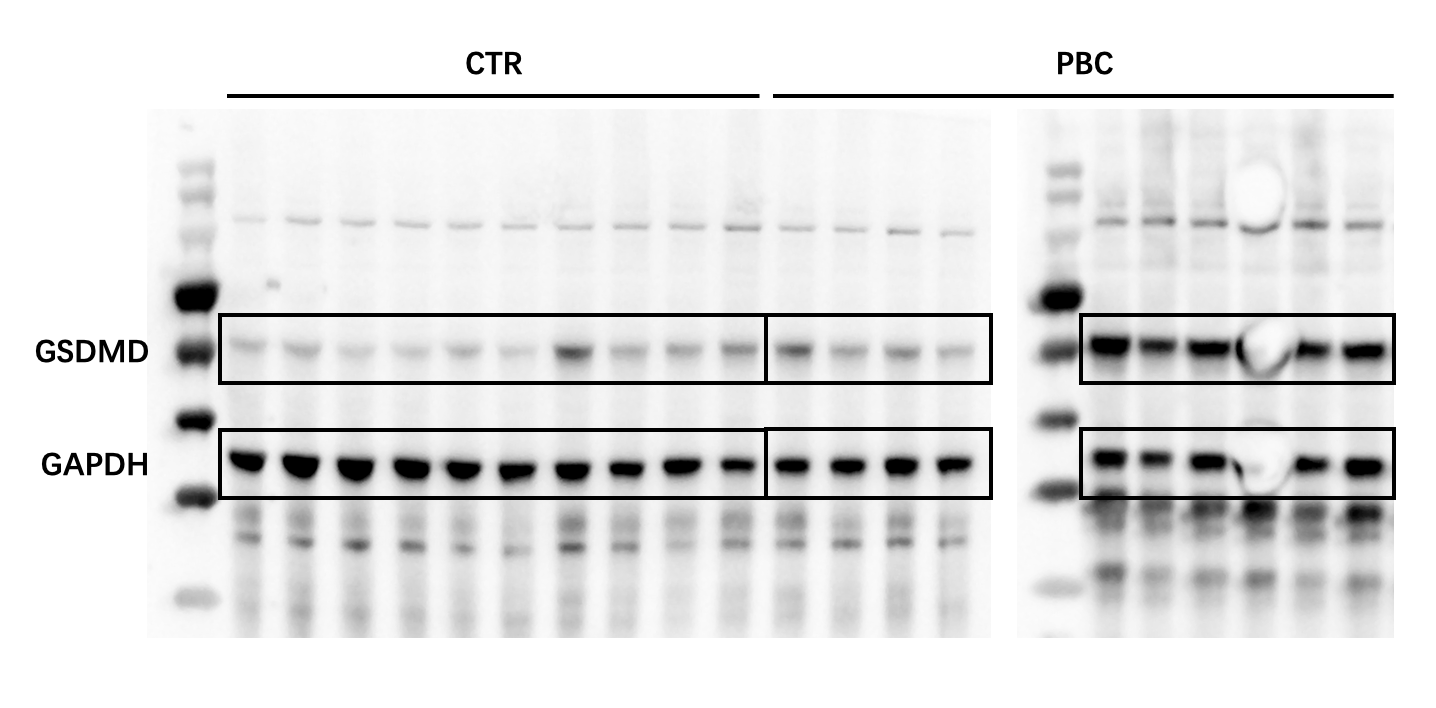


Table.S1 Antibodies Used in Immunofluorescent staining.

| Target antigen | Vendro | Category number |
| --- | --- | --- |
| CK-19 | Abcam | ab52625 |
| CD4 | Abcam | ab183685 |
| GSDMD | Abcam | ab219800 |
| Multiple immunofluorescence kit - Four colors TSA-Rab-275 | Panovue | 10079100020 |

Table.S2 Antibodies Used in Flow Cytometric Analysis.

| Target antigen | Fluorochrome | Vendro | Category number |
| --- | --- | --- | --- |
| CD45 | FITC | BioLegend | 157214 |
| CD11b | BV421 | BD Biosciences | 101235 |
| F4/80 | PE | BD Biosciences | 123110 |
| Ly6G | APC | BD Biosciences | 127614 |
| CD11c | APC | BD Biosciences | 117324 |
| CD206 | PE | BD Biosciences | 141720 |
